# Giant Renal Cell Carcinoma (RCC): A Case Report of Delayed Diagnosis and Management

**DOI:** 10.7759/cureus.42324

**Published:** 2023-07-23

**Authors:** Camila A Villacreses, Andrew B Herson, Mitchell C Boshkos, Bailey Beetz, Isaac Elkins, Joseph C Klink

**Affiliations:** 1 Urology, Lake Erie College of Osteopathic Medicine, Jacksonville, USA; 2 Urology, Lake Erie College of Osteopathic Medicine, Akron, USA; 3 Urology, Lake Erie College of Osteopathic Medicine, Bradenton, USA; 4 Urology, Lee Health, Fort Myers, USA

**Keywords:** rhabdoid, kidney cancer, parathyroid hormone-related peptide (pthrp), renal cell carcinoma (rcc), health care literacy

## Abstract

Renal cell carcinoma (RCC) is the most common type of kidney cancer. It typically presents with macroscopic hematuria, weight loss, and or a palpable flank mass. Diagnosis of this disease involves imaging techniques such as abdominal ultrasound and CT scans. Care for RCC can consist of ablation, tumor removal, nephrectomy, and systemic treatment options. Herein, we present a case of a 50-year-old Hispanic male with complaints of rectal bleeding and hematuria. Prior to admission, the patient had been informed twice about high suspicion of renal malignancy. Due to low health literacy and barriers to communication, he failed to understand the magnitude of his diagnosis. Subsequently, he underwent a resection of a considerable 22 cm x 13 cm x 13 cm RCC of his left kidney. This case highlights the need for effective patient health education to prevent emotional distress in patients with low health literacy.

## Introduction

Renal cell carcinoma (RCC) is a prevalent form of malignancy that accounts for many deaths each year, with about 81,000 new cases and 15,000 fatalities annually worldwide. Over the last two decades, there has been an increase of more than 30% in its occurrence. The reason for this rise is thought to be due to the more widespread use of radiological imaging techniques, leading to earlier diagnosis at an earlier stage. However, there has also been an increase in advanced disease and mortality rates [1]. 

The condition usually presents itself with one or more of the following symptoms: macroscopic hematuria, weight loss, or a palpable flank mass. The most common histologic subtype of RCC is clear cell RCC (ccRCC), although RCC represents a group of tumors that have a diverse range of histopathologic characteristics. The common organs to which metastasis from RCC occurs are the lungs, regional lymph nodes, bone, liver, adrenal glands, contralateral kidney, and brain [2]. Diagnosis relies most heavily on imaging including abdominal ultrasound and CT scan with and without contrast enhancement. On imaging, decreased attenuation suggestive of necrosis is often present. Urinalysis and various serologic testing are of lesser importance in diagnosis except in the setting of paraneoplastic syndromes associated with RCC. Treatment options include active surveillance, ablation, nephron-sparing tumor excision, nephrectomy, and systemic treatment including both chemotherapy and immunotherapy. Predictors of a poor prognosis include poor functional status and metastasis. 

Tumor size in RCC upon patient presentation is variable, however, large cohorts of patients in prior studies have revealed a typical mean tumor size of 5.3 cm (about 2.09 in), and it has been further demonstrated that the size of the RCC tumor is related to its malignant potential [3]. Giant RCC, a tumor with a volume of more than 1000 cc, is rare. Giant RCC is rarely seen because of its slow growth rate and the developments in advanced imaging techniques. A case report by Oviedo et al. found that the largest RCC in the world at 28 cm x 25 cm x 15 cm and a total volume of 10,500 cm3 was successfully resected [4]. Here, we present a case of a giant RCC of the left kidney, with a total weight of 2348 g (about 5.18 lbs.), and dimensions of 22 cm x 13 cm x 13 cm. 

## Case presentation

A 50-year-old Hispanic male with a past medical history of nephrolithiasis, voiding dysfunction, and alcohol abuse presented to the emergency department (ED) for complaints of rectal bleeding and hematuria. The patient reported that he had previously presented to a different hospital a few months ago with the same primary complaint. In his prior ED admission, he was found to be anemic, requiring a transfusion, and was made aware of a left renal mass and referred to urology, but failed to follow up. He later developed generalized weakness, fatigue, pain, and swelling in his left flank and left abdomen. 

On initial examination, he was afebrile and hemodynamically stable. Physical exam was positive for pallor, a soft and distended left midline/lower abdomen, and left flank tenderness to palpation (TTP). Additionally, normal bowel sounds were present. 

Initial vital signs on presentation were as follows: blood pressure of 119/77 mmHg, pulse rate of 90 bpm, temperature of 98.6°F, respiratory rate of 17 breaths per minute, and 100% oxygenation on room air. Laboratory tests included a complete blood count (CBC) and a basic metabolic panel (BMP). The CBC showed leukocytosis, anemia, and thrombocytosis (Table 1). 

**Table 1:** Complete blood count. WBC, white blood cell; RBC, red blood cell; MCV, mean corpuscular volume; MCH, mean corpuscular hemoglobin; RDW, red cell distribution width; MPV, mean platelet volume; L, liter; g/dL, grams per deciliter; μm3, cubic micrometers; pg/cell, picograms per cell; mm3, cubic millimeters; fL, femtoliters

	Laboratory value	Reference range
WBC count	11.2 × 10^9^/L	4.5-11.0 × 10^9^/L
RBC count	3.32 x 10^12^/L	4.3-5.9 x 10^12^/L
Hemoglobin	6.9 g/dL	13.5-17.5 g/dL
Hematocrit	24.6 %	41%-53%
MCV	74.1 μm^3^	80-100 μm^3^
MCH	20.8 pg/cell	25-35 pg/cell
RDW	20.5%	12-15%
Platelet count	590,000 mm^3^	150,000-400,000 mm^3^
MPV	9.1 fL	7.5-11.5 fL

A BMP showed an elevated blood urea nitrogen (BUN)/creatinine ratio, hyponatremia, hypercalcemia, and transaminitis with an obstructive pattern (Table 2).

**Table 2:** Basic metabolic panel. BUN, blood urea nitrogen; A/G ratio, albumin to globulin ratio; mg/dL, milligrams per deciliter; mL/min/1.73m2, milliliters per minute per 1.73 meters squared; mmol/L, millimoles per liter; g/dL, grams per deciliter; U/L, units per liter

	Laboratory value	Reference range
BUN	22 mg/dL	7-25 mg/dL
Creatinine	0.9 mg/dL	0.7-1.5 mg/dL
BUN/Creatinine ratio	23.2	6-22
Sodium	132 mmol/L	135-145 mmol/L
Potassium	4.9 mmol/L	3.5-5 mmol/L
Chloride	95 mmol/L	95-105 mmol/L
Carbon dioxide	27 mmol/L	22-32 mmol/L
Calcium	12.5 mg/dL	8.5-10.5 mg/dL
Protein, Total	7.4 g/dL	6.5-8.1 g/dL
Albumin	3.0 g/dL	3.5-5.0 g/dL
A/G ratio	0.7 g/dL	0.8-2.0 g/dL
Bilirubin, Total	0.9 mg/dL	0.0-1.2 mg/dL
Alkaline phosphatase	440 U/L	25-125 U/L
Aspartate transaminase	60 U/L	10-35 U/L
Gamma-glutamyl transferase	91 U/L	5-40 U/L

**Figure 1:**
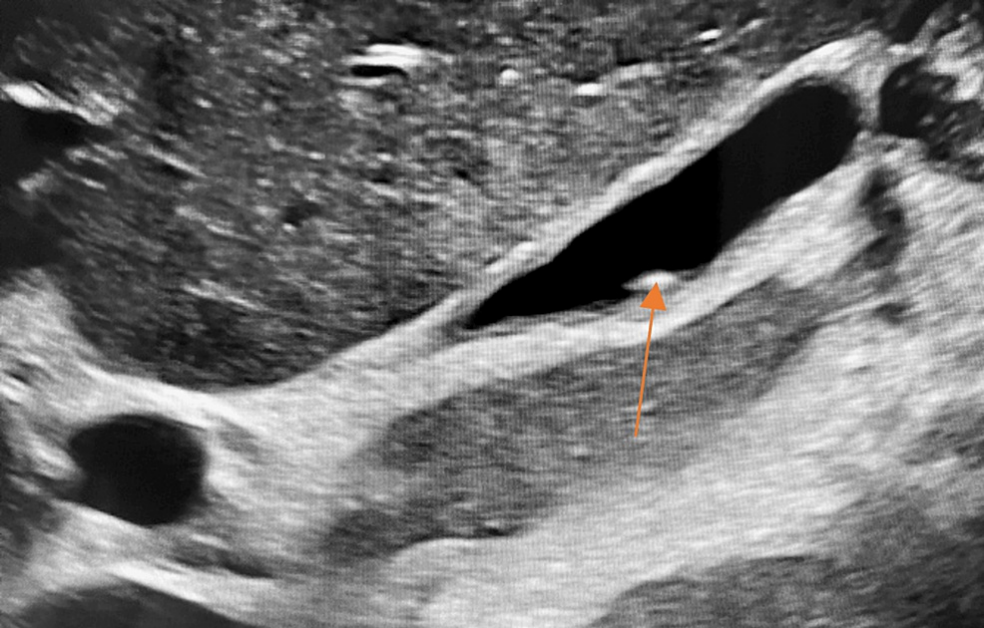
Abdominal ultrasound. Sagittal ultrasound view of the abdomen with a clear image of the gallbladder. A 6-mm stone can be appreciated within the gallbladder (orange arrow).

 Due to transaminitis with an obstructive pattern, an upper quadrant abdominal ultrasound (Figure 1) was ordered. 

**Figure 2:**
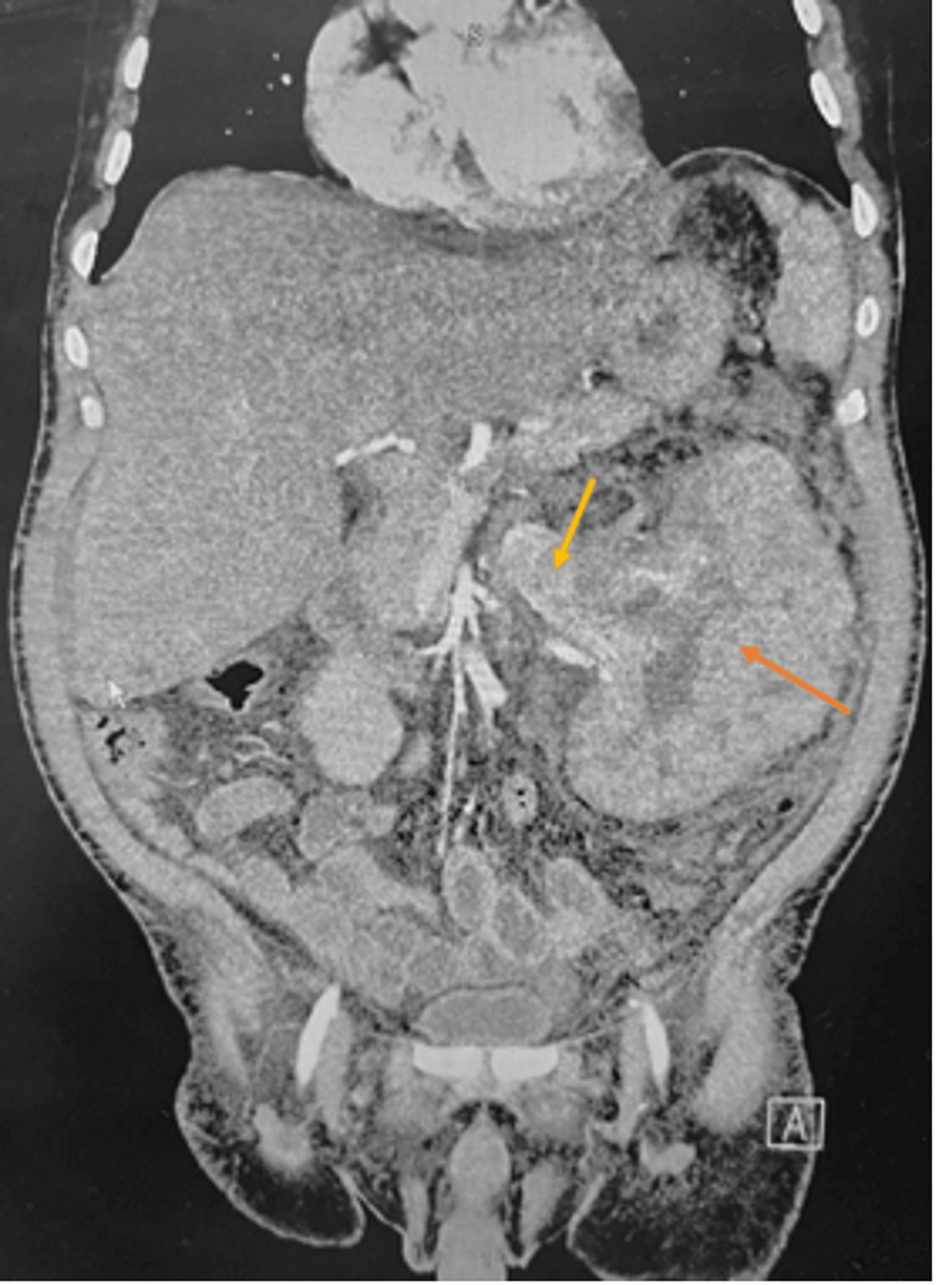
CTA of the abdomen and pelvis. CTA of the abdomen and pelvis demonstrating a large left renal mass (orange arrow) with the entire kidney in appearance and an enlarged left renal vein (yellow arrow). CTA, computed tomography angiogram

The abdominal ultrasound revealed a gallstone located within the gallbladder. A computed tomography angiogram (CTA) of the abdomen and pelvis was ordered due to the patient’s complaint of hematuria (Figure 2). 

**Figure 3:**
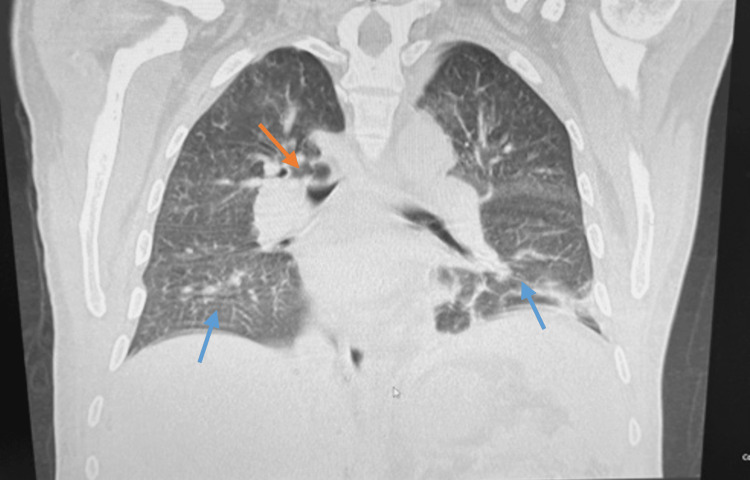
CT of the chest with contrast. CT of the chest (soft tissue) with contrast demonstrating an indeterminate roughly 7 mm nodule in the posterior right upper lobe (orange arrow). Large left and small right pleural effusions were also seen (blue arrows).

The abdominal and pelvic CTA revealed a large left renal mass and left renal vein enlargement, suggesting a likely tumor in the renal vein. Approximately 8 years ago, he was evaluated at another institution for intermittent gross hematuria and was informed of the left renal mass, however, the patient failed to follow up even though suspicion of malignancy was high. A CT of the chest with contrast was ordered to rule out distant metastatic disease, revealing an indeterminate 7 mm nodule in the posterior right upper lobe (Figure 3).

**Figure 4:**
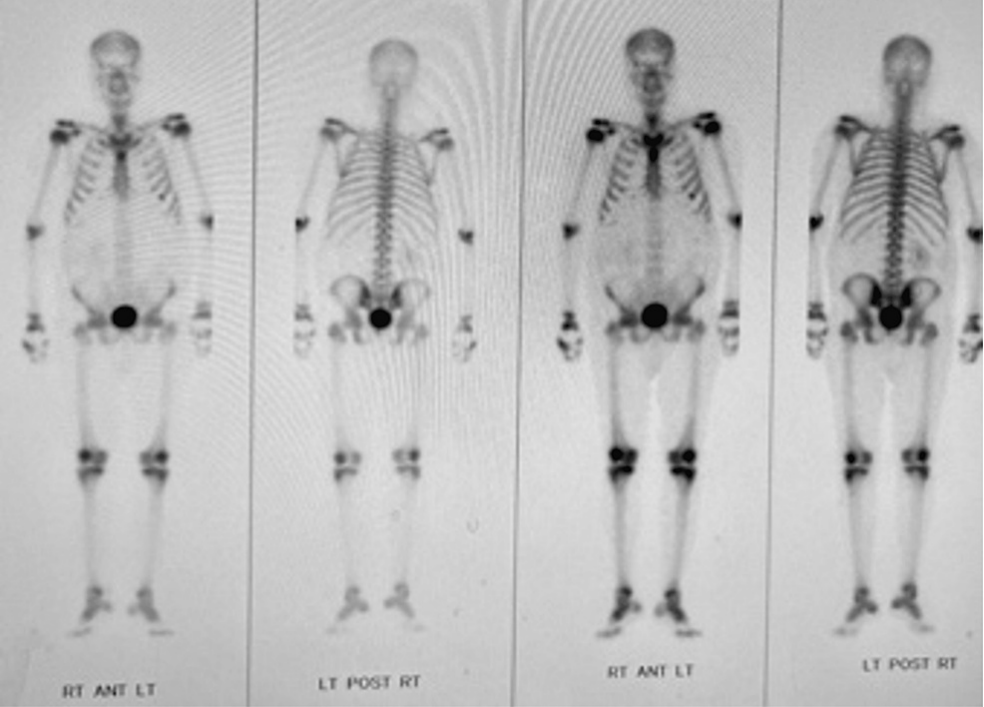
Bone scan. Bone scan of whole body showing no evidence of metastatic disease.

A bone scan was ordered for complete prognostic measure. The scan did not show evidence of metastatic disease (Figure 4). 

**Figure 5:**
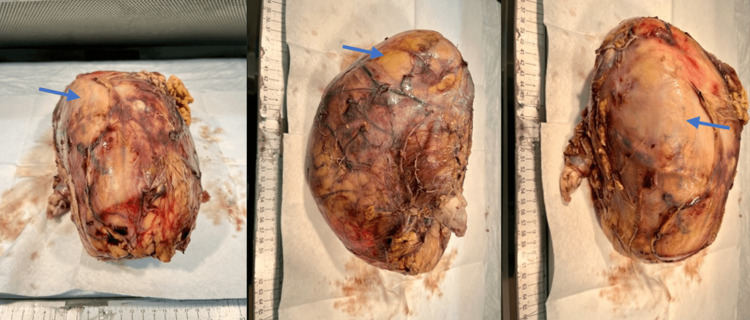
RCC of the left kidney. Giant renal cell carcinoma (RCC) of the left kidney (blue arrows), with a total weight of 2348 g (about 5.18 lbs.), and dimensions of 22 cm x 13 cm x 13 cm

A CT of the head without contrast was obtained due to the patient endorsing an acute onset of altered mental status (AMS). The study indicated no acute intracranial process. Thus, his AMS was concluded to be secondary to his hyponatremia, as well as the language barrier and his illiteracy. The patient subsequently underwent an open left radical nephrectomy with inferior vena cava (IVC) tumor thrombectomy, an open left adrenalectomy, and reconstruction of the IVC. The thrombus was located at the level of the left renal vein, advancing towards the IVC. A giant RCC was excised (Figure 5). 

Due to the patient’s low health literacy, language barrier, and limited education, he displayed a limited understanding of his overall status and was surprised by the gravity of his condition. He believed that his renal issues stemmed from a soccer injury he had many years ago. He stated that due to his illiteracy, he was unaware of the extent of his diagnosis. 

The patient was discharged home with a 2-week follow-up for surgical staple removal. On the day of discharge, the patient’s vital signs were as follows: blood pressure of 100/84 mmHg, pulse rate of 74 bpm, and respiratory rate of 17 breaths per minute. A CBC showed leukopenia and anemia (Table 3). 

**Table 3:** Complete blood count. WBC, white blood cell; RBC, red blood cell; MCV, mean corpuscular volume; MCH, mean corpuscular hemoglobin; MCHC, mean corpuscular hemoglobin concentration; RDW, red cell distribution width; MPV, mean platelet volume; L, liter; g/dL, grams per deciliter; μm3, cubic micrometers; pg/cell, picograms per cell; mm3, cubic millimeters; fL, femtoliters

	Laboratory value	Reference range
WBC count	4.3 × 10^9^/L	4.5-11.0 × 10^9^/L
RBC count	3.60 x 10^12^/L	4.3-5.9 x 10^12^/L
Hemoglobin	9.8 g/dL	13.5-17.5 g/dL
Hematocrit	30.1%	41%-53%
MCV	83.6 μm^3^	80-100 μm^3^
MCH	27.2 pg/cell	25-35 pg/cell
RDW	18.5%	12%-15%
Platelet count	284 mm^3^	150,000-400,000 mm^3^
MPV	9.9 fL	7.5-11.5 fL

A BMP showed hyperchloremia, hypocalcemia, hypoalbuminemia, and hypoproteinemia (Table 4).

**Table 4:** Basic metabolic panel. BUN, blood urea nitrogen; mg/dL, milligrams per deciliter; mL/min/1.73m2, milliliters per minute per 1.73 meters squared; mmol/L, millimoles per liter; g/dL, grams per deciliter

	Laboratory value	Reference range
BUN	7 mg/dL	7-25 mg/dL
Creatinine	0.69 mg/dL	0.7-1.5 mg/dL
BUN/Creatinine ratio	10.1	6-22
Sodium	136 mmol/L	135-145 mmol/L
Potassium	3.6 mmol/L	3.5-5 mmol/L
Chloride	107 mmol/L	95-105 mmol/L
Carbon dioxide	24 mmol/L	22-32 mmol/L
Calcium	6.9 mg/dL	8.5-10.5 mg/dL
Protein, Total	5.4 g/dL	6.5-8.1 g/dL
Albumin	2.4 g/dL	3.5-5.0 g/dL

## Discussion

In the United States, there are approximately 63,000 new cases of kidney cancer and around 14,000 deaths [2]. RCC, derived from renal tubular epithelial cells, makes up the majority of kidney cancers [2]. There are several subtypes of RCC. Clear cell RCC (ccRCC) makes up about 75% of RCC with the other subtypes papillary and chromophobe making up 15% and 5%, respectively [2]. The patient in our case was confirmed to have 22 cm x 13 cm x 13 cm ccRCC with rhabdoid differentiation and staging of pT3a N1M0. Inherited and sporadic forms of ccRCC are mostly caused by the loss of the tumor suppressor von Hippel-Lindau (VHL) gene [5]. VHL plays a key role in regulating the hypoxia-inducible factors (HIF)-hypoxia pathway, which when unchecked, allows the cell increased glucose metabolism, cell migration, vascular endothelial growth factor (VEGF) production, nutrient uptake, and dedifferentiation [5]. Even more specific to our patient, a loss in the tumor suppressor gene Brahma (BRM), is suggested to lead to the rhabdoid differentiation in ccRCC [6]. BRM is a part of the SWI/SNF complex responsible for chromatin remodeling and transcription regulation that can go awry in many types of malignancies, not just ccRCC with rhabdoid differentiation [7]. Unfortunately, ccRCC with rhabdoid differentiation is associated with poor prognosis [8]. Cancer-specific mortality has been found to be 40%-50% and median survival rates vary from 8 to 31 months [8]. With a metastasis rate of 70%, it is remarkable that our patient with long-standing ccRCC with rhabdoid differentiation had no evidence of metastasis upon further workup [8]. Previous studies have found this tumor type to be sized from 4 to 14 cm with an average of 9 cm [8]. Our patient’s tumor is unique in its presentation because it was large at 22 cm and was not associated with metastases, which is unlikely given rhabdoid differentiation. 

Additionally, ccRCC is known for causing multiple paraneoplastic symptoms. With up to 20% of patients affected, hypercalcemia is the most common. Half the time it is caused by the production of parathyroid hormone-related peptide (PTHrP) or parathyroid hormone (PTH) and the other half because of bony metastasis [9]. Our patient exhibited an elevated calcium of 12.5 mg/dL upon presentation which decreased to below normal range at 6.9 mg/dL two weeks after resection. With a negative bone scan, there is good evidence our patient was exhibiting his hypercalcemia from either PTHrP or PTH production. Interestingly, a previous surgical resection of a PTHrP-secreting hepatocellular carcinoma (HCC) elicited hypocalcemia postoperatively [10]. While no mechanism has been elicited, it seems plausible that the endogenous suppression of PTH by hypercalcemia could cause a delay in PTH production from the parathyroid gland leading to postoperative hypocalcemia in our patient and the previously mentioned HCC patient. 

The ccRCC is classically known for an increase in erythropoietin (EPO) which can occasionally cause polycythemia [9]. However, our ccRCC patient was anemic, which has actually been shown to be more common than polycythemia in ccRCC patients [9]. This is proposed to be due to the anemia of chronic disease, but our patient had substantial hematuria, so this undoubtedly played a role in his anemic state (hemoglobin of 6.9 g/dL) and the need for a blood transfusion [9]. It was unfortunate that this patient’s care had been delayed to a point where a blood transfusion was necessary. 

The patient presented above faced health disparities throughout his disease course which resulted in delayed or incomplete care. Health disparities are defined as preventable differences in health outcomes in certain populations including but not limited to age, race, ethnicity, income, education, and location [11]. Why these differences exist is a topic of active research, but it is evident there is an intrinsic link between health disparities and health literacy [12]. Health literacy is the ability of a patient to have access to care, understand their disease, and make informed decisions about their treatment [13]. It has been estimated that 80 million Americans have low health literacy [14]. Certain populations are disproportionally more suspectable to low health literacy, and these populations are inherently related to those that struggle with health disparities [15]. 

The patient in this case had multiple risk factors contributing to his vulnerability to health disparities including having a limited education and being a non-native English speaker. As mentioned in the case presentation above, the patient had an 8-year history of hematuria and flank symptoms. He had been informed by healthcare professionals multiple times to follow up with care for his concerning condition. By the time he presented in the ED many years after the initial symptoms, his RCC had grown to a massive size and was causing him significant morbidity. It is possible given the patient’s demographic that he perhaps did not receive clear information to be able to understand his illness at the initial presentation, did not have the resources to access care, or there was some other social or economic barrier preventing him from seeking care. Likely there was a combination of all these aspects at play and subjecting him to health disparities. His low health literacy contributed to his inability to be a self-advocate and prevented his prompt care. Having low health literacy has been shown to be associated with poorer health outcomes and poorer healthcare utilization [15]. The patient here is a prime example of how delayed care due to low health literacy can lead to multiple ED visits, prolonged disease course, increased morbidity, and riskier surgery. 

It is evident from this case discussion that it should be a top goal of the healthcare institution to strive to improve the health literacy of the population at large and minimize health disparities. Current research is being conducted to create and continually evolve strategies to improve health literacy. Here are several effective strategies that physicians and other healthcare providers can implement in their practice now: use medical jargon-free vocabulary, convey only a few key points per visit (so as to not overwhelm the patient), utilize interpreters when necessary, describe disease and treatment with pictures and diagrams, and use the teach-back method to establish patient understanding [16]. These simple changes at the individual level can have incredible impacts on the patients directly by improving health outcomes and decreasing morbidity, on healthcare as an institution by decreasing cost and increasing efficiency, and on society by combatting systemic inequalities.

## Conclusions

Giant RCC, while considered rare due to its slow growth rate and early detection by advanced imaging techniques, can be encountered in cases such as this. Its occurrence in recent years has increased due to the use of imaging techniques to identify the disease. This case highlights the need for clinical outreach to educate patients on common health diagnoses and how to navigate their personal health. Thus, it is important that healthcare teams are effective and prepared in communicating with patients in a manner that ensures comprehension. While this may place a burden on healthcare teams to source the services of translators, it may prevent exacerbation or mortality of the disease. 
